# Agénésie de la valve pulmonaire: à propos d'un cas chez une sénégalaise de 24 ans

**DOI:** 10.11604/pamj.2014.18.76.3250

**Published:** 2014-05-24

**Authors:** Affangla Désiré Alain, Leye Mohamed, Dia Aliou Amadou, Ndiaye El Hadj Mohamed, Aw Fatou, Bazolo Georges Antoine, Kane Abdoul

**Affiliations:** 1 Hôpital Saint Jean de Dieu, Thiès, Senegal; 2 UFR des sciences de la santé de l'université de Thiès. BP 43 Thiès, Senegal; 3 Hôpital Barthimée, Thiès, Senegal; 4 Pédiatre. Centre Hospitalier Régional de Louga, Sénégal; 5 Hôpital Général Grand Yoff Dakar, Senegal

**Keywords:** Agénésie, valve pulmonaire, cardiopathie congénital, Agenesis, pulmonary valve, congenital heart disease

## Abstract

L'agénésie de la valve pulmonaire est une cardiopathie congénitale rare (6/3000 cardiopathies congénitales). La tolérance clinique et le pronostic de la forme avec communication inter ventriculaire dépendent de la compression des voies respiratoires par l'artère pulmonaire dilatée. Nous rapportons un cas chez une sénégalaise de 24 ans.

## Introduction

L'agénésie des valves pulmonaires est une cardiopathie congénitale rare (6/3000 cardiopathies congénitales) définie par une absence totale ou une hypoplasie sévère des sigmoïdes pulmonaires. Dans la majorité des cas, elle s'associe à d'autres anomalies, en particulier une communication inter ventriculaire isolée. La tolérance clinique et le pronostic dépendent particulièrement de la compression des voies respiratoires par l'artère pulmonaire dilatée.

## Patient et observation

A.N, sénégalaise de 24 ans, présentant une dyspnée d'effort et des lipothymies depuis l’âge d 12 ans. L'examen clinique retrouve un état de maigreur avec IMC à 15, une cyanose, un hippocratisme digital avec une SpO2 à 66% en air ambiant. A l'auscultation, un souffle systolique rude éjectionnel 4-5/ 6 frémissant associé à un souffle diastolique au foyer pulmonaire. Il n'y a pas de signes périphériques d'insuffisance cardiaque.

**Figure 1:**
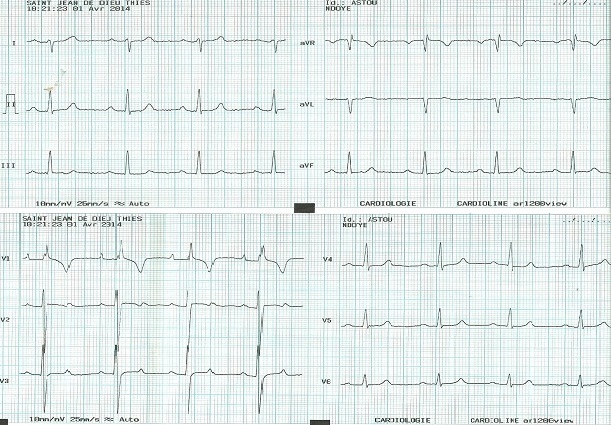
ECG 12 dérivations s'inscrivant en rythme sinusal avec une déviation axiale droite à 120%, avec une hypertrophie auriculaire et ventriculaire droite

L’électrocardiogramme s'inscrit en rythme sinusal avec une déviation axiale droite à 120%, avec une hypertrophie auriculaire et ventriculaire droite de type systolique (Figure 1).

**Figure 2:**
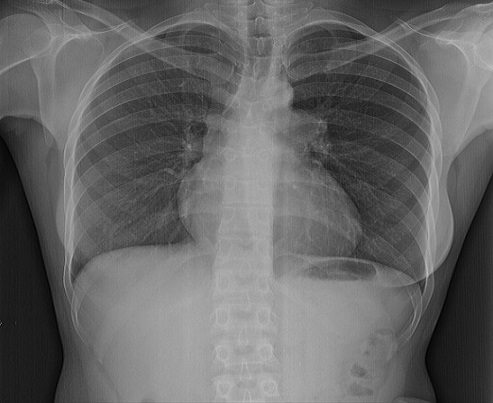
Radiographie télécœur de face montrant une cardiomégalie modérée (index cardio- thoracique à 0, 53), une convexité de l'arc moyen gauche, une vascularisation pauvre en périphérie. Dilatation de la partie proximale de l'artère pulmonaire gauche

A la radiographie télécœur de face on note une cardiomégalie modérée avec un index cardio- thoracique à 0, 53, une convexité de l'arc moyen gauche, une vascularisation pauvre en périphérie et une artère pulmonaire proximale dilatée (Figure 2).

**Figure 3:**
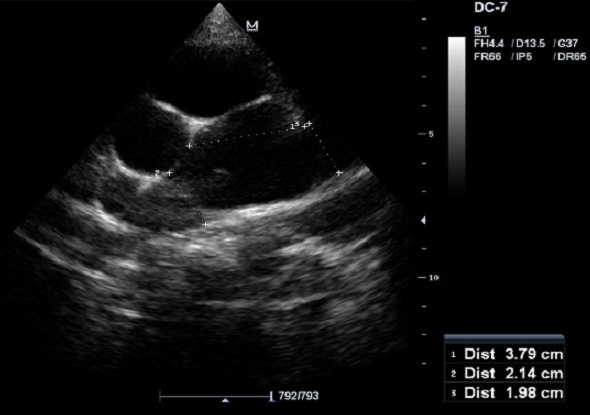
Echographie cardiaque, Incidence petit axe para sternale montrant l'agénésie de la valve pulmonaire avec dilatation ectasique du tronc et des branches pulmonaires

**Figure 4:**
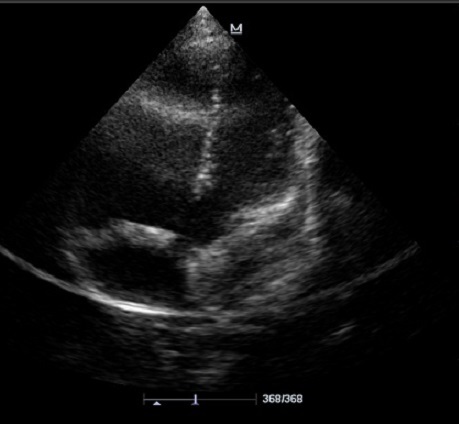
Échographie cardiaque en incidence 5 cavités apicale, montrant une large communication inter ventriculaire (CIV) sous une aorte dextroposée, une dilatation modérée du ventricule droit

L’écho-doppler cardiaque (Figure 3, Figure 4) montre des cavités droites modérément dilatées, des cavités gauches de bonne taille avec une bonne fonction systolique des ventricules. Il existe une large communication inter ventriculaire (CIV) sous aortique avec shunt bidirectionnel à travers la CIV. On note une Dextroposition aortique avec continuité mitro -aortique. Il existe une agénésie de la valve pulmonaire avec accélération à 4m/s du flux pulmonaire antérograde. Le tronc et les branches de l'artère pulmonaires sont dilatés. La bifurcation pulmonaire est bien dégagée, les deux branches pulmonaires naissent du tronc de l'artère pulmonaire et il n'y a pas de sténose sur les deux branches pulmonaires. Le canal artériel est fermé. Il y avait une insuffisance pulmonaire modérée à moyenne.

**Figure 5:**
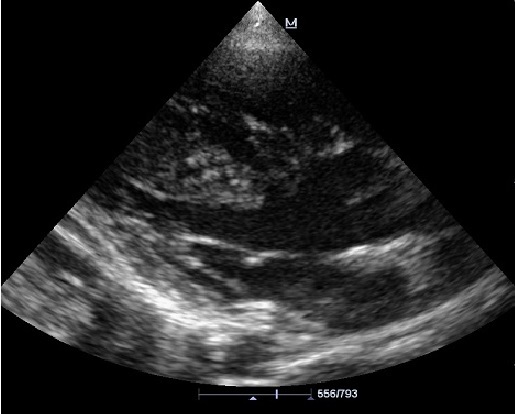
Angioscanner thoracique en coupe axiale montrant une dilatation bilatérale des artères pulmonaires. Noter le discret effet de masse sur les bronches souches

L'angioscanner thoracique (Figure 5) montre sur une coupe axiale une dilatation importante du tronc de l'artère pulmonaire et de ses branches avec un discret effet de masse sur les bronches souches.

L'exploration fonctionnelle respiratoire(EFR) par spirométrie montre une restriction pulmonaire modérée avec une capacité vitale lente à 70% de la valeur théorique.

## Discussion

L'agénésie des valves pulmonaires est une malformation cardiaque rare. Cette malformation associe une hypoplasie restrictive de l'anneau pulmonaire, une agénésie des valves pulmonaires et une dilatation anévrysmale des artères pulmonaires. Lorsque l'agénésie de la valve pulmonaire est associée à une communication inter ventriculaire (CIV), cette malformation est considérée par certains comme une variante de la tétralogie de Fallot dont elle représenterait 5% des formes [1, 2].

La tolérance clinique est le plus souvent mauvaise associant des signes respiratoires par compression bronchique secondaire aux artères pulmonaires anévrysmales à des signes d'insuffisance cardiaque dans l'agénésie pulmonaire sans communication inter ventriculaire [3]. Cette malformation peut être relativement bien tolérée et vue chez l'adulte dans la forme avec CIV, comme le cas que nous rapportons.

Chez notre patiente, la cyanose, l'hippocratisme digital et le souffle systolique de sténose pulmonaire font évoquer de tétralogie de Fallot plus fréquemment vue chez l'adulte [3]. Cependant l'absence de malaises anoxiques et de squatting nous ont fait douter de ce diagnostic.

A la radiographie télécœur, l'hypo vascularisation pulmonaire associée à la convexité de l'arc moyen gauche et la dilatation de la partie proximale de l'artère pulmonaire gauche (Figure 2) sont évocateurs du diagnostic de l'agénésie pulmonaire.

Le diagnostic est fermement établi par l’écho doppler cardiaque qui montre le micro développement des sigmoïdes pulmonaires, une dilatation importante de l'artère pulmonaire et de ses branches, un large infundibulum, une fuite pulmonaire importante, la CIV permettent d’établir le diagnostic.

La tolérance clinique et le pronostic dépendent principalement du développement du ventricule droit [4] et de la compression bronchique par l'artère pulmonaire dilatée [5]. Notre patiente est symptomatique depuis l’âge de 12 ans mais elle ne présente pas de signes majeurs de compression bronchique (bronchite répétitive et détresse respiratoire avec wheezing) [6] et le ventricule droit a un développement normal. L'angioscanner thoracique peut être d'un apport capital dans l'exploration des malformations cardiaques [5–8] et montre chez notre patiente une dilatation bilatérale des artères pulmonaires comprimant discrètement les bronches souches (Figure 5).

L'exploration fonctionnelle respiratoire (EFR) par spirométrie peut contribuer à apprécier le retentissement respiratoire [1, 2] et montre chez notre patiente une restriction pulmonaire modérée avec une capacité vitale lente à 70% de la valeur théorique.

Un traitement chirurgical, comprenant une ouverture de l'anneau, une plastie des branches [11–15] est envisagé chez notre patiente.

## Conclusion

L'agénésie des valves pulmonaires avec communication inter ventriculaire est une cardiopathie congénitale à révélation le plus souvent tardive. L’échocardiographie-Doppler est l'examen de référence pour faire le diagnostic. Cependant l'angioscanner thoracique et la spirométrie contribuent qualitativement au diagnostic.
